# Activating Endogenous Condylar Stem Cells to Enhance TMJ Repair

**DOI:** 10.1177/00220345251363561

**Published:** 2025-10-16

**Authors:** T. Tuwatnawanit, N. Anthwal, A.S. Tucker

**Affiliations:** 1 Centre for Craniofacial and Regenerative Biology, Faculty of Dentistry, Oral and Craniofacial Sciences, King’s College London, London, UK; 2 Department of Conservative Dentistry and Prosthodontics, Faculty of Dentistry, Srinakharinwirot University, Wattana, Bangkok, Thailand

**Keywords:** fibrocartilage, signal pathways, temporomandibular joint disorders, osteoarthritis, regeneration, stem cell niche

## Abstract

The temporomandibular joint (TMJ) plays a critical role in the daily activities of mastication and communication, with disorders of the TMJ significantly impairing quality of life. Temporomandibular disorders (TMDs) are highly prevalent, presenting a pressing need for regenerative therapies. The TMJ’s key components—condyle, TMJ disc, and glenoid fossa—are crucial for proper function; however, the limited self-repair capability of these tissues makes managing TMJ pathology particularly challenging. Emerging research in animal models has emphasized the importance of fibrocartilage stem/progenitor cells (FCSCs) located in and around the superficial layers of the condyle. Lineage tracing of condylar FCSCs in vivo has identified subpopulations with different contributions to growth and homeostasis, providing potential targets for regenerative therapies. In addition to the FCSCs, niche-supporting cells have been recently identified in the superficial layers of the condyle, further highlighting the complex cellular environment of the TMJ. Several signaling pathways, including Wnt, Hedgehog, and Notch, play pivotal roles in establishing cell fate in the developing and growing TMJ and have been additionally implicated in both the control of FCSC populations and progression of TMDs. Recent research has used this understanding of the signaling pathways involved in the creation of the joint to stimulate the endogenous stem cells/FCSCs of the adult in vivo, leading to enhancement of regenerative capacity in mouse, rat, rabbit, and porcine injury and disease models. Manipulation of signaling pathways has been combined with advanced bioengineering techniques, providing scaffolds to allow controlled dispersal of activators and inhibitors. Such advances in understanding the triggers and molecular mechanisms that control TMJ FCSCs, combined with improved targeting of specific signaling pathways, have opened new avenues for regenerative therapies. These insights have begun to be leveraged in the development of novel hydrogel-based injectable regenerative therapeutic approaches to not only alleviate symptoms but also promote true regeneration of TMJ structures.

## Introduction

The temporomandibular joint (TMJ) comprises 2 primary skeletal components: the mandibular condylar head and the glenoid fossa (or mandibular fossa) of the squamous portion of the temporal bone (Fig. 1A). A fibrous articular disc, known as the TMJ disc, acts as a cushion between these articulating surfaces (Wadhwa and Kapila 2008; Anthwal and Tucker 2020). Proper TMJ function is essential for the complex jaw movements required for daily activities such as mastication and communication (Wadhwa and Kapila 2008; Bender et al. 2018). Temporomandibular disorder (TMD) is an umbrella term encompassing a wide variety of disorders that affect the structural, functional, or physiological dysregulations of the TMJ (Suzuki and Iwata 2015). TMDs affect 34% to 38% of the world population, with women at higher risk than men (Minervini et al. 2023; Zieliński et al. 2024). The high prevalence of TMJ dysfunction across all age groups underscores the need for more effective diagnostic criteria and therapeutic strategies (Rongo et al. 2021; Zieliński et al. 2024). TMJ osteoarthritis (TMJOA) is a major chronic degenerative condition characterized by progressive cartilage degradation and abnormal subchondral bone remodeling (Goldring and Goldring 2016; Yao et al. 2023; Feng et al. 2024). Notably, meta-analyses and systematic reviews indicate TMJOA affects between 5.2% and 17.4% of patients with TMDs according to the diagnostic criteria used (Valesan et al. 2021). TMJOA leads to severe joint pain, functional impairment, and malocclusion, significantly affecting patients’ overall health and quality of life (Ning et al. 2022). Current treatments for TMD often provide only symptomatic relief or surgical treatment, rather than addressing the underlying causes or promoting true regeneration of the joint (Xu et al. 2022; Feng et al. 2024). Up to 20% of TMD patients needed definitive therapy, and as much as 5% needed surgical interventions (de Souza et al. 2012). These cohorts might benefit from the translation of stem cell therapy into the clinics. Given the complexity of TMJ structure and function, as well as its limited capacity for self-repair, identifying mechanisms that can promote repair and regeneration is crucial (Xu et al. 2022). One avenue is to use our understanding of how the TMJ forms and grows during embryonic and postnatal development, and how stem cell populations are established and controlled, to learn how to build new TMJ tissues in the adult. Interestingly, developmental processes that drive formation in the embryo are often recapitulated in repair mechanisms, offering a blueprint for regenerative strategies (Goldfield and Coppens 2020; Chatzeli et al. 2021).

**Figure 1.**
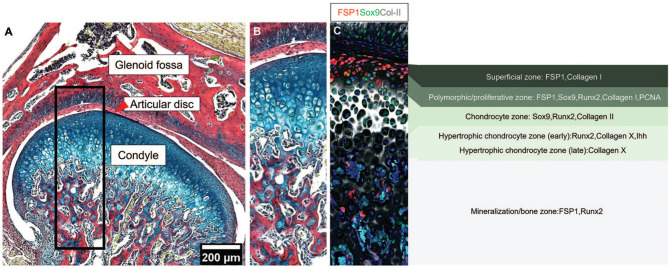
TMJ structure (**A**, **B**). Picrosirius red–Alcian blue trichrome staining of the TMJ in CD1 mice at postnatal day 28. The boxed area highlights a zoomed-in view of the glenoid fossa, articular disc/TMJ disc, and condyle, showing the layers of the condyle in Trichrome staining in B and immunofluorescence staining for FSP1 (red), Sox9 (green), Collagen II (gray), and DAPI (blue) in (**C**). The mature mandibular condyle is structured into 4 zones: a superficial zone (expressing FSP1, collagen I), a polymorphic/proliferative zone (expressing FSP1, Sox9, Runx2, Collagen I, PCNA), a chondrocyte zone (expressing Sox9, Runx2, Collagen II), and a hypertrophic chondrocyte zone (early hypertrophic chondrocytes expressing Runx2, Collagen X, Ihh, and late hypertrophic chondrocyte expressing Collagen X) (Shibukawa et al. 2007), with cells moving as a conveyer belt through the zones as they differentiate into the mineralization zone forming bone (expressing FSP1, Runx2). TMJ, temporomandibular joint.

In development, the TMJ forms through complex and tightly regulated temporospatial processes, with the condyle acting as a growth center creating the organized layered pattern of the condyle and contributing to growth of the dentary bone (Shibata et al. 2008; Liang et al. 2015). The mature mandibular condyle is structured into 4 zones, which are essential for mandibular growth, joint function, and the unique remodeling capacity of the condylar cartilage. Unlike articular cartilage of the long bones, condylar cartilage contributes directly to bone elongation during development. The zones are (1) a superficial zone (fibrocartilage articular surface, expressing FSP1 and collagen I); (2) a polymorphic/proliferative zone of proliferative cells, generating cells down to the jaw (expressing FSP1, Sox9, Runx2, Collagen I, PCNA); (3) a chondrocyte zone, where cells differentiate into chondrocytes (expressing Sox9, Runx2, Collagen II); and (4) a hypertrophic chondrocyte zone (early hypertrophic chondrocyte expressing Runx2, Collagen X, Ihh, and late hypertrophic chondrocyte expressing Collagen X) with cells moving as a conveyer belt through the zones as they differentiate into the mineralization zone forming bone (expressing FSP1, Runx2) (Shibukawa et al. 2007; Tsutsumi-Arai et al. 2023) (Fig. 1B, C). During development, distinct populations of fibrocartilage stem cells (FCSCs) have recently been shown to be established in the condyle, providing potential targets for regenerative therapies (Embree et al. 2016; Tuwatnawanit et al. 2025).

This review explores the key signaling pathways that regulate TMJ development, growth, and homeostasis. Importantly, many of the signaling pathways that play a role in development are also implicated in degenerative TMJ disorders, particularly TMJOA, providing links between development, disease, and repair. Specifically, we will focus on how signaling pathways interact with endogenous stem cell populations and their niche in the condyle, exploring their potential for developmental bioengineering and regeneration of damaged TMJ tissues. By studying TMJ biology and its developmental processes, we can unlock new ways to manipulate FCSCs to promote joint repair.

### Signaling Pathways in TMJ Morphogenesis and Growth

A number of signaling pathways have been shown to be active during TMJ development and growth, helping to coordinate formation of the joint, establish patterns, and control proliferation. These pathways work in concert to ensure proper joint function, growth, and adaptability. Here we will focus on the Wnt, Hedgehog, and Notch pathways.

The Wnt signaling pathway plays a pivotal role in the formation of synovial joints in the embryo, and pathway constituents are expressed in the murine condyle from initiation (Guo et al. 2004; Chen et al. 2017). During early postnatal TMJ development, *Axin2*, a readout of Wnt signaling activity, was highly expressed in the TMJ, suggesting roles in tissue growth and differentiation (Chen et al. 2017; Tuwatnawanit et al. 2025), while *Lgr5*, a receptor involved in Wnt signaling, was expressed in the disc (Ruscitto et al. 2023). However, during postnatal development, Wnt activity diminished in the superficial layers of the condyle, aligning with the reduced need for cartilage formation and growth in adulthood (Chen et al. 2017; Tuwatnawanit et al. 2025). In keeping with this, Wnt pathway inhibitors were expressed in condyle progenitor clusters by single cell RNA sequencing (scRNAseq) (Wang et al. 2024). While Wnt activity reduces in the superficial layers postnatally, it is maintained in the differentiating cells of the TMJ, with nuclear β-catenin, a key downstream mediator of canonical Wnt signaling, maintained in mature chondrocytes and hypertrophic chondrocytes (Wang et al. 2024).

Hedgehog (Hh) signaling plays a pivotal role in both the embryonic development and postnatal homeostasis of the TMJ, with components of the pathway expressed throughout morphogenesis (Shibukawa et al. 2007; Purcell et al. 2009). During embryonic development, loss of Indian hedgehog (Ihh) signaling in Ihh^-/-^ mice prevented the initiation and separation of the TMJ disc from the condyle, resulting in loss of the disc (Shibukawa et al. 2007). The patterning of the condyle was also affected, with reduced proliferation of the polymorphic layer (Shibukawa et al. 2007). Overexpression of Ihh in *Wnt1-Cre;pMes-Ihh* mice also led to TMJ defects, with delayed cell differentiation of the condyle and loss of the fossa, highlighting the importance of establishing the correct balance of Hh signaling (Yang et al. 2016). Mutations in *Gli2*, a downstream component of the Hh pathway, has been linked to human mandibular hypoplasia, while mouse *Gli2* knockouts led to disc defects, similar to those observed in *Ihh* mutants (Purcell et al. 2009; Berolacini et al. 2012). In addition to morphogenesis, Hh signaling is important for postnatal growth. *Gli1*, a readout of Hg signaling widely used to identify Hh-responsive cells, is expressed in the superficial layers of the condyle and the underlying subchondral bone postnatally (Bechtold et al. 2019; Wang et al. 2024). In keeping with this, the postnatal loss of *Ihh* led to defects in TMJ cartilage maturation and bone formation (Ochiai et al. 2010).

Notch signaling plays an important role in regulating chondrocyte differentiation. Notch 1 and 2 remain cytoplasmic in developing cartilage but shift to the nucleus as cells enter the hypertrophic zone (Hosaka et al. 2013). In the TMJ, Notch activity (using a fluorescent Notch reporter) was evident from the early stages of morphogenesis, with cells later localized within the superficial zone of the condylar cartilage and the TMJ disc (Ruscitto et al. 2020; Tosa et al. 2023). At early stages, Notch signaling appeared to inhibit differentiation, while at later stages it stimulated differentiation, highlighting context-dependent effects and a dual role in regulating cartilage (Hosaka et al. 2013).

In addition to these pathways, scRNA-seq of human developing condyles has highlighted the Fgf pathway (Zhu et al. 2023). *Fgfr1*, *2*, and *3* isoforms are expressed in the TMJ during development (Rice et al. 2003). Analysis of *Fgfr3* mutant mice has suggested Fgf signaling acts upstream of Hh signaling to coordinate TMJ morphogenesis, with mutants displaying disruption in the normal layering pattern of the condyle (Yasuda et al. 2012).

### Characterization of Fibrocartilage Stem/Progenitor Cells in the TMJ

The TMJ articular surfaces are protected by fibrocartilage (Temenoff and Mikos 2000). Fibrocartilage is specialized for load bearing and is composed of fibrous and cartilaginous components, contributing to its ability to withstand both tensile and compressive force (Singh and Detamore 2009; Stocum and Roberts 2018). Fibrocartilage, lining the TMJ articular surfaces, has unique molecular features compared with hyaline or elastic cartilage, notably high levels of collagen I and the expression of fibroblast-specific protein 1 (FSP1)/S100A4 (Delatte et al. 2004; Park et al. 2015; Tuwatnawanit et al. 2025). Retention of thymidine analogues such as EdU and BrdU is widely used as a hallmark of stem cells (Sottocornola and Lo Celso 2012). These markers are incorporated into DNA during the S-phase of the cell cycle and become diluted with each subsequent cell division. EdU/BrdU-positive label-retaining cells (LRCs) have been shown to reside within the superficial layers that form the articular surface in both mouse and rat, suggesting that these layers house an endogenous stem cell population of FCSCs (Embree et al. 2016; Wang et al. 2024; Tuwatnawanit et al. 2025) (Fig. 2A–D). FCSCs have been identified within condylar cartilage in rats, mice, rabbits, and humans (Embree et al. 2016; Bi et al. 2020). In rats, FCSCs harvested from the superficial zone of the condyle could be expanded in vitro and had the potential to differentiate into a range of cell types, including chondrocytes, adipocytes, and osteoblasts (Embree et al. 2016). Similarly, human FCSCs derived from human condylar cartilage have been shown to exhibit distinct stemness, with a notable capacity for chondrogenic differentiation (Bi et al. 2020; Fan et al. 2021). In comparison to the chondrocytes of the condyle, the FCSCs had a greater capacity to propagate and an enhanced differentiation potential in vitro (Embree et al. 2016). The discovery of FCSCs in the condyle has garnered increasing research interest, particularly regarding their potential to be stimulated for therapeutic applications.

**Figure 2.**
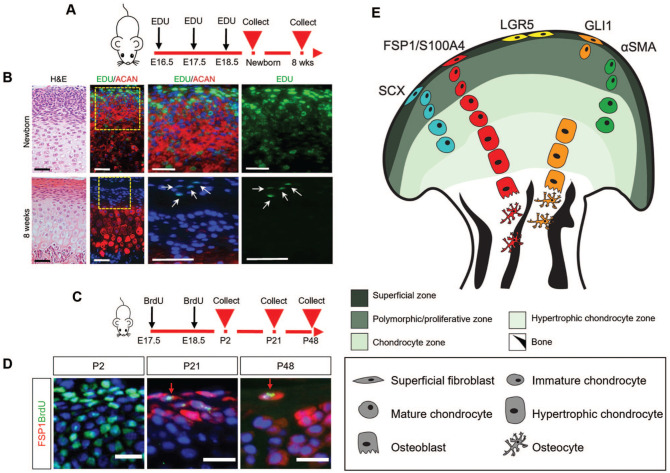
Tracing endogenous stem and niche cell populations in the TMJ condyle. (**A**) EdU was administered to Sprague–Dawley pregnant rats at E16.5, E17.5, and E18.5. EdU-positive LRCs were chased in newborns and at 8 wk. (**B**) H&E and immunohistochemistry for EdU (green), aggrecan/ACAN (red), and DAPI (blue). The newborn group showed that EdU was successfully incorporated into the nuclei of condylar cartilage cells. As condylar growth and cell proliferation occurred, EdU labeling became diluted. In 8 wk, EdU-positive LRCs, which are slow-cycling cells indicating stem cell characteristics, were only located in the superficial zone of the condyle. Arrows indicate the EdU-positive LRCs (green) in the superficial zone of the condyle. Scale bar, 50 µm. (**C**) BrdU was administered to CD1 mice at E17.5 and E18.5. BrdU-positive LRCs were chased in P2, P21, and P48. (**D**) Immunofluorescence staining for BrdU (green), FSP1 (red), and DAPI (blue). BrdU-positive cells were detected in most of the condylar cells at P2, confirming BrDU label uptake (green). Condylar cells proliferated rapidly, leading to a significant reduction in BrdU-positive cells by P21 and P48. By P48, all BrdU-positive LRCs in the upper layers of the condyle expressed FSP1, indicating that FSP1 marks a novel stem/progenitor cell population in the TMJ. Arrows indicate FSP1-expressing cells (red) co-stained with BrdU-positive LRCs (green). Scale bar, 20 µm. (**E**) Lineage tracing schematic of Scx (blue), FSP1 (red), LGR5 (yellow), Gli1 (orange), and α-SMA (green) labeled cells and their contributions from the superficial zone, polymorphic/proliferative zone, chondrocyte zone, hypertrophic chondrocyte zone, and bone. α-SMA, α–smooth muscle actin; BrdU, 5-Bromo-2′-deoxyuridine; EdU, 5-ethynyl-2′-deoxyuridine; FSP1, fibroblast-specific protein 1; H&E, haematoxylin and eosin; LRC label-retaining cell; Scx, scleraxis; TMJ, temporomandibular joint. (A, B) Adapted with permission from Embree et al. (2016). (C, D) Adapted with permission from Tuwatnawanit et al. (2025).

To map these FCSC populations in the condyle, lineage tracing studies have been instrumental in identifying and understanding the location and potential of in vivo stem/progenitor cells involved in the postnatal TMJ (Fig. 2E, Table). The expression of a number of genes, including *Acta2*, *Scleraxis (Scx)*, *Gli1*, and *FSP1*, have been identified as markers of these putative stem/progenitor cells and their progeny (Embree et al. 2016; Ma et al. 2021; Chen et al. 2022; Lei et al. 2022; Tuwatnawanit et al. 2025) (Fig. 2E). The expression and location of these markers do not completely overlap, suggesting that FCSCs in the condyle may present a heterogeneous population with differing potential (Fig. 2E). Lineage tracing of α–smooth muscle actin (α-SMA) (product of *Acta2*) in *α-SMACreERT2;Ai9* mice demonstrated that undifferentiated α-SMA–positive cells near the superficial zone of the condyle could give rise to mature Col2a1-positive chondrocytes in the condylar cartilage zone (Embree et al. 2016). The *ScxCre^ERT2^;R26R^tdTomato^* mouse model demonstrated that *Scx*-lineage cells in the superficial layers of the condyle could differentiate into prechondroblasts and chondrocytes postnatally, leading to TMJ expansion during postnatal development (Ma et al. 2021). Long-term lineage tracing following EdU injection in *Gli1-LacZ* and *Gli1Cre^ERT2^;tdTomato* mice demonstrated that LRCs partially co-localized with *Gli1*-positive cells in the superficial zone of the condyle (Wang et al. 2024). This small subset of Gli1-positive cells exhibited self-renewal capacity and could differentiate into mature and hypertrophic chondrocytes (Wang et al. 2024). *Gli1*, therefore, serves as a marker for both stem and progenitor cells that continuously support cartilage turnover during the postnatal period (Wang et al. 2024). In addition to contributing to the top layers of the condyle, *Gli1*-positive cells from the subchondral bone contributed to the osteoblastic lineage and co-expressed osteogenic markers (Chen et al. 2022; Lei et al. 2022) (Fig. 2E).

**Table.** Characteristics of Fibrocartilage Stem Cells in the Condyle.

Stem/niche Cell Population	Lineage Tracing Mouse Line	Induction and Chase Period	Terminal Differentiated Cell Type and Location	Characteristic/ Roles in TMJ Condylar Cartilage Processes
α-SMA	*α-SMACreERT2;Ai9*	• Tamoxifen P16 • Collect P18 (2 d after injection) and P31 (15 d after injection)	Undifferentiated α-SMA–positive cells near the superficial zone of the condyle gave rise to mature Col2a1-positive chondrocyte progeny in the condylar cartilage zone (Embree et al. 2016)	Unknown
Scleraxis	*ScxCre^ERT2^;R26R^tdTomato^*	• Tamoxifen P3 • Collect at P4, P9, P33, P60	Scx-lineage cells in the fibrous layer of the condyle differentiated into prechondroblasts and chondrocytes (Ma et al. 2021)	Unknown
Gli1	*Gli1Cre^ERT2^;tdTomato*	• Tamoxifen P3.5 • Collect 2 d and 7 after injection (Wang et al. 2024)	Gli1-positive cells in the superficial zone of the condyle could differentiate into mature and hypertrophic chondrocytes In vitro experiment showed multipotential differentiation of Gli1-positive cells (Wang et al. 2024)	In a TMJOA mouse injury model, the *Gli1*-lineage was activated and differentiated into hypertrophic chondrocytes, leading to depletion of the stem cell pool (Wang et al. 2024)
		• Tamoxifen 1 mo for 3 times in 3 d • Collect 1 d, 1 mo, 3 mo, 6 mo, 9 mo, 12 mo after injection (Lei et al. 2022)	Gli1 lineage from the subchondral bone contributed to osteoblastic lineage and co-expressed osteogenic markers (Lei et al. 2022)	TMJOA mouse injury model led to Hh signaling activation; Gli1-positive cell derivatives were shown to undergo excessive expansion and heightened osteogenic differentiation (Lei et al. 2022)
		• Tamoxifen P0.5 • Collect 3 d, 1 wk, 1 mo after injection (Chen et al. 2022).	Gli1-positive cells at the chondro-osseous junction contributed to trabecular bone (Chen et al. 2022)	Gli1-positive osteogenic progenitors contributed to condylar fracture repair (Chen et al. 2022)
FSP1/S100A4	*FSP1-Cre;mTmG*	• Noninducible • Collect E17.5, P14, P21, P56, P63	FSP1 labeled the superficial articular surface of the postnatal TMJ and gave rise to all condylar cartilage and bone FSP1 population overlapped with *Axin2, Scx* and *Gli1 mRNA* (Tuwatnawanit et al. 2025)	FSP1 expression was negatively regulated by canonical Wnt activity in vivo Ablation of FSP1-positive cells resulted in TMJOA (Tuwatnawanit et al. 2025)
Lgr5	*Lgr5^EGFPcre+/-;^ROSA/tdTomato*	• Tamoxifen P1, P2, P3 • Collect P180	*Lgr5*-expressing secretory cells appear to act as niche cells in the superficial layer of the condyle, creating a Wnt inhibitory niche (Ruscitto et al. 2023)	Lgr5-positive secretory cells regulate chondrocyte identity and maintain chondroprogenitors (Ruscitto et al. 2023)

α-SMA, α–smooth muscle actin; FSP1, FSP1, fibroblast-specific protein 1; Hh, Hedgehog; TMJ, temporomandibular joint; TMJOA, TMJ osteoarthritis.

Interestingly, the expression of *Scx* and *Gli1* mRNA in the superficial layers of the condyle has been shown to overlap with FSP1 (Tuwatnawanit et al. 2025). FSP1 labels the superficial articular surface of the postnatal TMJ. The *FSP1-Cre;mTmG* mouse model has shown that FSP1-positive cells give rise to the entire condylar skeleton, suggesting that FSP1 may serve as a marker for putative stem/progenitor cells in the TMJ (Tuwatnawanit et al. 2025). Similar to *Gli1*, a subset of FSP1-positive cells is label retaining (Fig. 2C, D). FSP1 expression is induced postnatally in the mouse, suggesting this is the time point when the FCSC populations are established. Interestingly, expression increased significantly at weaning, suggesting mechanical force may play a role in establishing the FCSC population (Tuwatnawanit et al. 2025). A similar, mechanically induced, postnatal stem cell population has been suggested in the junctional epithelium around the tooth (Yuan et al. 2023). Ablation of the FCSCs in vivo in the mouse, using diphtheria toxin driven in *FSP1* or *Gli1* cells, led to severe disruption of condyle patterning and failed growth, highlighting the essential role of these cells (Wang et al. 2024; Tuwatnawanit et al. 2025).

Recent advances in scRNAseq have provided further insights into the cellular and molecular landscape of the TMJ and the character of the FCSCs. Mouse condyle analysis at the single-cell level confirmed *Gli1* as a stem cell marker in the condyle and highlighted further markers to explore, such as *Smoc1* (Wang et al. 2024).

### FCSC Interactions with Signaling Pathways

The establishment of the FCSC population postnatally in the mouse correlates with the loss of Wnt signaling in this region, suggesting links between signaling pathways and establishment and maintenance of the condyle FCSCs. To test this, the overexpression of Wnt signaling in the superficial layers using *FSP1-Cre;βcatGOF* mice led to a disruption of the superficial layers of the condyle and loss of expression of FSP1 (Tuwatnawanit et al. 2025). Interestingly, postnatal *Axin2* lineage tracing, using the *Axin2-CreERT2;tdTom* mouse, showed that FSP1-positive cells were in the *Axin2*-lineage (Tuwatnawanit et al. 2025). Early Wnt signaling may therefore be important for initiation of the fibrocartilage layer and FCSC population. *Lgr5*-expressing secretory cells appear to act as niche supporting cells in the superficial layer of the condyle, helping to create a Wnt-inhibitory niche (Ruscitto et al. 2023). Loss of this Wnt inhibition led to unstable chondrocytes with osteoblast-like properties (Ruscitto et al. 2023). Similarly, knockout of the canonical Wnt inhibitor *sclerostin* (*SOST*), and thereby activation of Wnt signaling, led to a significant reduction in FCSCs in the superficial zone of the condyle (Embree et al. 2016). Adding SOST to FCSCs in vitro sustained cartilage formation while limiting bone formation, confirming that a regulated inhibitory signal is necessary to preserve the FCSC pool and promote balanced cartilage regeneration (Embree et al. 2016). Although the Wnt-inactivated environment is crucial for preserving the FCSC niche, Wnt activation becomes essential when FCSCs divide, exit their niche, and begin differentiation (Tosa et al. 2023; Wang et al. 2024). In keeping with this, the loss of canonical Wnt function using *Gli1-Cre^ERT2^;β-catenin^fl/fl^* mice led to an inhibition of hypertrophic cartilage differentiation and bone formation (Wang et al. 2024). Thus, the balance of Wnt levels depends on tissue specificity. While a low level of Wnt is required in fibrocartilage layers, a high level of Wnt is required in bone.

*Gli1*-positive cells have also been shown to be responsive to Wnt/β-catenin signaling with the addition of a Wnt inhibitor in vitro leading to downregulation of both proliferation and osteogenic differentiation of *Gli1*-positive cells (Chen et al. 2022). Notch signaling has also been implicated in controlling the condyle FCSCs, with Notch activity localized to the superficial zone in mice postnatally. In adult mice, the activation of the Notch pathway in superficial cells in vitro promoted differentiation of the FCSCs to bone and cartilage, whereas blocking Notch suppressed the bone fate, suggesting a role for Notch signaling in defining FCSC cell fate decisions (Ruscitto et al. 2020).

Another potential signaling pathway involved in control of FCSCs in postnatal mice is *insulin-like growth factor 1 (Igf1)* (Bi et al. 2023). Igf1 was shown to regulate TMJ cartilage growth and metabolism by influencing the differentiation, proliferation, and apoptosis of FCSCs (Bi et al. 2023). The deletion of *Igf1* in FCSCs led to a disorganized fibrocartilage layer, and a decreased number of condylar cartilage cells (Bi et al. 2023).

### Dysregulation of Signaling Pathways Leads to TMJOA

In disease and injury conditions, many signaling pathways associated with development and growth have been shown to be altered, either as a consequence of disease or as a driver (Fig. 3A, B). This has been studied in a range of animal models, where TMJOA has been induced by injury (such as partial discectomy) or inflammation (tumor necrosis factor–α [TNF-α] injection), and from patient tissue removed during surgical treatment for TMJOA.

**Figure 3.**
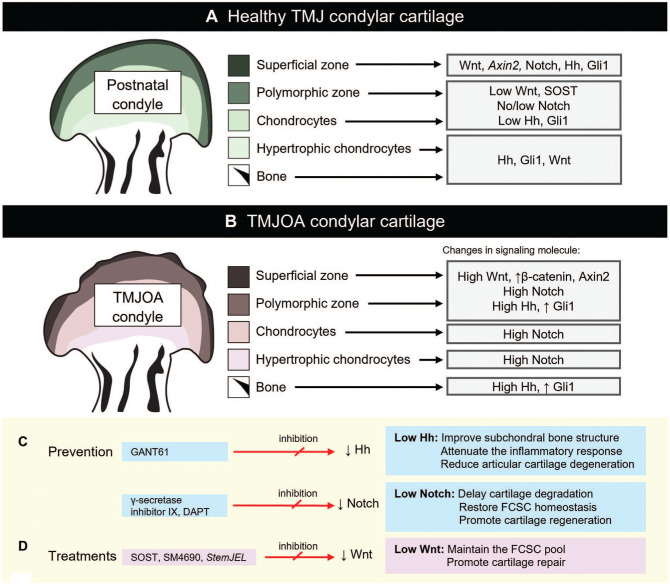
Schematic representation of Wnt, Hh, and Notch pathway expression in TMJ condylar cartilage during homeostasis and disease. (**A**) Healthy TMJ condylar cartilage during postnatal homeostasis. (**B**) TMJOA in condylar cartilage. (**C**) Potential prevention with treatment administered immediately after injury or within 1 d using an intra-articular injection targeting Hh and Notch signaling pathways. (**D**) Potential therapeutic strategies with treatment applied 1 wk to 1 mo postinjury using intra-articular injection targeting the Wnt signaling pathway. TMJ, temporomandibular joint; TMJOA, TMJ osteoarthritis.

Using a partial discectomy (PD)–induced TMJOA mouse model, *Gli1-*positive cell derivatives were shown to undergo excessive expansion and heightened osteogenic differentiation in response to damage, favoring commitment to the osteoblastic lineage (Lei et al. 2022). This injury model led to Hh signaling activation and an uneven distribution of osteoblastic lineage differentiation, resulting in abnormal subchondral bone remodeling and accelerated TMJOA progression (Lei et al. 2022). Using a similar injury mouse model, the *Gli1* lineage was activated and differentiated into hypertrophic chondrocytes, leading to depletion of the stem cell pool (Wang et al. 2024). Interestingly, the application of TNF-α to induce an inflammatory TMJOA phenotype inhibited FCSC proliferation and survival with loss of *Gli1*-positive cells (Bi et al. 2021), highlighting that different models of TMJOA may have different effects on FCSCs.

Importantly, patients with more severe osteoarthritis scores had a higher number of β-catenin–positive cells in their TMJ fibrocartilage compared with patients with less severe osteoarthritis, revealing that the severity of disease is linked to higher levels of canonical Wnt activity (Embree et al. 2016). To identify whether changes in Wnt could drive TMJOA, several studies have manipulated the Wnt signaling pathway postnatally in animal models. Overactive canonical Wnt signaling was shown to disrupt fibrocartilage homeostasis by depleting the FCSC pool and destabilizing chondrocyte phenotypes, leading to fibrocartilage degeneration and contributing to TMJOA (Embree et al. 2016; Ruscitto et al. 2023). Studies using conditional activation of β-catenin have also demonstrated TMJOA-like phenotypes (Hui et al. 2018). In *FSP1-Cre;βcatGOF* mice, constitutive Wnt/β-catenin signaling specifically in the FSP1-expressing cells resulted in severe osteoarthritic changes (Tuwatnawanit et al. 2025). Loss of Wnt inhibitors, such as *SOST*, also led to osteoarthritic changes in the condylar head (Embree et al. 2016). Collectively, these animal manipulation studies highlight that enhanced canonical Wnt signaling contributes to the progression of TMJOA.

Elevated Notch signaling has also been observed in TMJOA (Saito and Tanaka 2017; Luo et al. 2018; Ruscitto et al. 2020; Liu et al. 2023). Following localized administration of TNF-α to induce TMJOA, Notch-positive Col II/Runx2-positive cells were observed in the superficial zone and at the cartilage–bone interface (Ruscitto et al. 2020). Sustained Notch signaling in a Notch gain of function using *Col2a1Cre;tetO-NICD1;Rosa-rtTA^f/+^* mouse model led to apoptosis of articular chondrocytes, alteration in extracellular matrix synthesis, increased cartilage degradation, cartilage fibrosis, and development of severe osteoarthritis-like pathology (Liu et al. 2015). Together, these findings reveal that dysregulation of Hh, Wnt, and Notch signaling pathways affect FCSCs, thereby contributing to the pathogenesis of TMJOA.

### Leveraging Cellular Signaling to Stimulate Endogenous FCSCs for Repair

The recent advances in our understanding of how FCSC populations are established, maintained, and altered in disease and injury models present promising new avenues for manipulating FCSCs in vivo by directing signaling pathways to trigger regeneration and reestablish homeostasis (Fig. 3C, D).

As highlighted above, the balance of canonical Wnt signaling is essential for maintaining fibrocartilage integrity in the TMJ. Suppressed Wnt signaling supports FCSC homeostasis and preserves cartilage structure, whereas overactivation disrupts these processes, leading to fibrocartilage degeneration and the progression of TMJOA. Given the pivotal role of Wnt signaling in regulating FCSCs and its dysregulation in TMJOA conditions, therapeutic interventions targeting this pathway have shown promising outcomes (Fig. 3D). Specifically, the application of exogenous sclerostin (SOST) has demonstrated the ability to maintain the FCSC pool and promote cartilage repair. In a rabbit disc perforation–induced TMJOA model, SOST injections effectively rescued the disease phenotype compared with the contralateral TMJ (Embree et al. 2016). The intra-articular injection of SM04690 (commercially known as Lorecivivint), a Wnt pathway inhibitor, has been shown to protect against condylar cartilage degeneration and mitigate abnormal subchondral bone remodeling in rat and rabbit models of PD-induced TMJOA (Hua et al. 2022; Gill et al. 2023). This protective effect was attributed to several mechanisms, including an increase in the FCSC pool within the superficial zone of the condyle, enhanced chondrogenic differentiation of FCSCs, inhibition of chondrocyte hypertrophy, and suppression of proteolytic enzymes that degrade the articular cartilage matrix (Hua et al. 2022). In addition, SM04690 treatment reduced β-catenin expression in TMJ condylar cartilage and protected chondrocytes from TNF-α–induced inflammatory responses (Hua et al. 2022). Recently, *StemJEL*, an innovative injectable hydrogel therapy combining 2% high-molecular-weight hyaluronic acid and recombinant SOST protein, proved effective in restoring cartilage homeostasis, preserving chondrocyte identity, and improving joint function by reducing canonical Wnt activity (Ruscitto et al. 2023). This treatment has yielded positive results in both rabbit and mini-pig models of TMJOA, demonstrating cartilage restoration levels comparable with those of healthy controls (Ruscitto et al. 2023). These findings highlight the Wnt signaling pathway as a compelling therapeutic target for addressing TMJOA and advancing regenerative treatment strategies.

As an alternate avenue, pharmacologic inhibition of Hh signaling in *Gli1*-positive osteogenic progenitors using a direct antagonist of *Gli1/2*-mediated transcription, the Gli-antagonist 61 (GANT61), was shown to alleviate TMJOA by improving subchondral bone structure, attenuating the inflammatory response, and reducing articular cartilage degeneration (Lei et al. 2022). This finding underscores the critical role of Hh signaling in the pathological progression of TMJOA, particularly through its regulation of *Gli1*-positive progenitors. By targeting this signaling pathway, the research suggests a promising therapeutic approach to mitigate cartilage degradation and other degenerative changes associated with TMJOA (Lei et al. 2022) (Fig. 3C).

As Notch activation plays a critical role in driving the cartilage-to-bone transformation of FCSCs in the context of TMJOA, Notch inhibitors such as γ-secretase inhibitor IX or N-[N-(3,5-difluorophenacetyl-L-alanyl)]-(S)-phenylglycine t-butyl ester (DAPT) have been shown to significantly reduce the expression of *Notch1*, *Runx2*, and *Osteocalcin* in FCSCs (Ruscitto et al. 2020). This suppression effectively inhibited osteogenic differentiation and blocked cartilage-to-bone transformation in vitro (Ruscitto et al. 2020). The injection of DAPT after a discectomy operation in mice reduced Notch signaling activation (Notch receptor, ligand and downstream gene) and delayed the onset of cartilage degradation while mitigating TMJOA severity (Luo et al. 2018). Targeting Notch signaling may help restore FCSC homeostasis and promote cartilage regeneration while mitigating inflammation-induced joint damage. Therefore, Notch inhibitors hold significant therapeutic promise for treating TMJOA by preserving the condyle’s morphology (Fig. 3C).

TNF-α activation has been shown to disrupt the osteogenic and chondrogenic differentiation of FCSCs; however, modulating the TNF-α/NF-κB signaling pathway can mitigate these regulatory effects (Bi et al. 2021). Inhibition of TNF-α signaling reduced NF-κB transcript levels and restored the chondrogenic potential of FCSCs, facilitating cartilage repair in TMJOA (Bi et al. 2021). Furthermore, in vivo intra-articular treatment with etanercept, a TNF-α inhibitor, effectively rescued TMJ cartilage degeneration, mitigating growth retardation and alleviating the osteoarthritis phenotype in anterior disc displacement–induced TMJOA (Bi et al. 2021). These findings reveal a novel therapeutic mechanism by which TNF-α/NF-κB inhibition enhances the chondrogenic capacity of FCSCs, promoting cartilage regeneration in TMJOA. Similarly, Fostamatinib (Fos), a potential clinical drug for rheumatoid arthritis that can target NF-κB, has demonstrated therapeutic efficacy in treating unilateral anterior crossbite-induced TMJOA in rats via intra-articular injection (Zhang et al. 2025). How this treatment affects the FCSCs of the joint and its interaction with other signaling pathways is an interesting area for future analysis.

## Conclusions and Perspectives

A deeper understanding of TMJ development, homeostasis, and the regulatory roles of signaling pathways in controlling FCSCs is essential for advancing therapeutic strategies (Cardoneanu et al. 2022). Exploring the potential of endogenous stem cells for TMJ regeneration and repair offers promising avenues for future treatment. By integrating insights from developmental biology, disease pathology, and stem cell–based therapies, this review highlights the potential for innovative approaches to address TMJOA and improve patient outcomes. Although signaling pathways are known to play a role in TMJ development and pathogenesis, and studies on the interactions between β-catenin and other signaling molecules during osteoarthritis are ongoing, significant challenges remain (Tosa et al. 2023; Yao et al. 2023; Feng et al. 2024). The intricate interplay between the Wnt, Hh, Notch, and other signaling pathways during both TMJ development and osteoarthritis progression remains largely unexplored. The interaction of these key signaling pathways within a complex gene regulatory network is seldom investigated in TMJOA disease models. A clear understanding of the contribution of signaling pathways and their interaction network could identify potential targets for treating TMJOA. However, many of these pathways play dual roles in the condyle, turning off in some areas while remaining on in others. The manipulation of pathways, therefore, needs to be targeted, to avoid disruption of normal function. The development of such targeted therapeutics, which could affect specific populations of cells, will be important for creating safe and effective therapeutic strategies in translational medicine. The signaling pathways are also dynamic and change over time during disease progression. Recent research using animal models with different intervention timelines after injury highlights the complexity of picking the timing when to apply therapeutics. In addition, selecting the appropriate animal model will be essential for ensuring successful translation to human clinical scenarios (Herring 2003). This review is the first to integrate insights from embryonic and postnatal development, TMJ homeostasis, and endogenous stem cell–based regenerative models, offering a holistic perspective on TMJ biology. This integrative framework paves the way for innovative treatments that aim not only to alleviate TMJ dysfunction but also to restore its structure and function effectively.
